# Cytogenetic and molecular diagnostic testing associated with prenatal and postnatal birth defects

**DOI:** 10.1002/bdr2.1648

**Published:** 2020-03-01

**Authors:** Stela Z. Berisha, Shashi Shetty, Thomas W. Prior, Anna L. Mitchell

**Affiliations:** ^1^ Center for Human Genetics University Hospitals Cleveland Medical Center Cleveland Ohio; ^2^ Department of Pathology Case Western Reserve University, University Hospitals Cleveland Ohio; ^3^ Department of Genetics and Genome Sciences Case Western Reserve University, University Hospitals Cleveland Ohio

**Keywords:** chromosomal/SNP array, fluorescence in situ hybridization (FISH), genetic testing, germline, karyotype, methylation, multiplex ligation‐dependent probe amplification (MLPA), next‐generation sequencing (NGS), PCR, postnatal defects, prenatal defects, Sanger sequencing

## Abstract

Genetic testing is beneficial for patients and providers when in search of answers to medical problems related to the prenatal or early postnatal period. It can help to identify the cause or confirm a diagnosis associated with developmental delay, intellectual disability, dysmorphic features, heart defects, multiple malformations, short stature, stillbirth, neonatal death, or fertility problems. Genetic testing can be used to rule out single‐gene or chromosome abnormalities. Different diagnostic cytogenetic and molecular genetic techniques are applied in clinical genetics laboratories, from conventional ones to the state of the art chromosomal microarrays and next‐generation sequencing. Each of the genetic techniques or methods has its strengths and limitations, however different methods complement each‐other in trying to identify the genetic variation(s) responsible for a medical condition, especially the ones related to birth defects.

## 1 INTRODUCTION TO THE NECESSITY FOR GENETIC TESTING

Genetic testing is required or necessary to be performed in various circumstances, often during the prenatal or early postnatal period. There are different scenarios when medical personnel will turn to genetic testing in search of answers for their patients, especially when there are problems of early growth and development such as developmental delays, failure to thrive, intellectual disability, dysmorphic features, multiple malformations, heart defects, short stature, ambiguous genitalia, and so on. Genetic testing can be valuable to couples or families who wish to understand the causes of stillbirth, neonatal death or fertility problems. Genetic testing may also rule out chromosome abnormalities in pregnancy, particularly in cases of pregnancy in women of advanced age (>35 years old). Oftentimes genetic testing is performed or desired when there is a known family history of either a chromosomal abnormality or a monogenic Mendelian disorder (single‐gene disorder).

The genetic methods are used to look into the human genome as one would be looking inside a book. Looking into the structural and chromosome abnormalities by cytogenetic methods is very similar to searching whether a book has all its chapters and pages and that it has them in the correct order. Fluorescence in situ hybridization is similar to looking for missing paragraphs within book pages. Molecular genetic techniques will take it one step further and provide insight on small disease‐causing structural genomic variations, which is to say looking inside phrases for missing words or misspellings.

This review will cover the most conventional and state of the art diagnostic genetic techniques that assess genome imbalances for chromosome abnormalities and/or single‐gene defects, will cover their possible outcomes, provide an in‐depth discussion of results, and lastly mention the implications and limitations. The focus of this review is to describe genetic tests related to constitutional (germline) abnormalities. The acquired (somatic) genetic abnormalities fall outside of its scope, however many of the genetic tests described herein are applicable to somatic changes as well.

## 2 CONVENTIONAL CYTOGENETIC ANALYSIS METHODS

### 2.1 Chromosome analysis

Most chromosome abnormalities are prenatally lethal or not compatible with life. It is estimated that approximately 15% of pregnancies result in miscarriage and chromosomal abnormalities account for about 50–60% of cases, in particular those that occur in the first trimester.Hassold & Hunt, 2001; Nagaoka, Hassold, & Hunt, 2012). Chromosomal abnormalities associated with first trimester miscarriages can be divided into two major categories: numerical and structural abnormalities. Examples of numerical abnormalities include trisomy of chromosome 16 (the most common one, associated with approximately 16% of miscarriages due to chromosomal abnormalities), monosomy X, triploidy (3n/69), or tetraploidy (4n/92) (Table 1). For an estimated incidence of aneuploidies in humans at different prenatal or postnatal stages please refer to the review article by Nagaoka et al. (2012). Additionally, chromosomal abnormalities have been observed in about 5% of stillbirths and perinatal deaths, 0.5–1% of live births and account for about 5% of cases when couples experience recurring multiple spontaneous abortions (carriers of balanced rearrangements). There is a correlation between the number of genes missing or existing in extra copies and the chance of an embryo or fetus not surviving. Chromosomes 13, 18, and 21 carry the lowest number of genes compared to other chromosomes (except for the Y chromosome) and therefore trisomies 13, 18, and 21 are better tolerated and a small percentage of them make it to term compared to other chromosome trisomies or monosomies. For the other autosomal chromosomes, trisomy or monosomy status is deleterious and incompatible with life (Table 1). Failure of chromosomes to separate normally during cell division(s) results in the generation of cells with missing or extra chromosomes and these could be either whole or parts of chromosomes (Hassold & Hunt, 2001; Mefford & Eichler, 2009).

**Table 1:** Abnormalities associated with prenatal or postnatal defects and their respective disorders

Common prenatal and postnatal chromosomal aneuploidies		
Name	Karyotype	Other names/disorder
Trisomy 21	47,XX,+21 or 47,XY,+21	Down syndrome
Trisomy 18	47,XX,+18 or 47,XY,+18	Edwards syndrome
Trisomy 13	47,XX,+13 or 47,XY,+13	Patau syndrome
Monosomy X	45,X	Turner syndrome
XXX	47,xxx	Triple X
XXY	47,XXY	Klinefelter syndrome
XYY	47,XYY	YY syndrome/Jacob's syndrome
Other fetal lethal chromosomal aneuploidies (except for monosomy X, all other chromosomal monosomies are incompatible with life)		
Trisomy 1 (47,XX,+1 or 47,XY,+1)	Trisomy 2 (47,XX,+2 or 47,XY,+2)	Trisomy 3 (47,XX,+3 or 47,XY,+3)
Trisomy 4 (47,XX,+4 or 47,XY,+4)	Trisomy 5 (47,XX,+5 or 47,XY,+5)	Trisomy 6 (47,XX,+6 or 47,XY,+6)
Trisomy 7 (47,XX,+7 or 47,XY,+7)	Trisomy 8 (47,XX,+8 or 47,XY,+8)	Trisomy 9 (47,XX,+9 or 47,XY,+9)
Trisomy 10 (47,XX,+10 or 47,XY,+10)	Trisomy 11 (47,XX,+11 or 47,XY,+11)	Trisomy 12 (47,XX,+12 or 47,XY,+12)
Trisomy 14 (47,XX,+14 or 47,XY,+14)	Trisomy 15 (47,XX,+15 or 47,XY,+15)	Trisomy 16 (47,XX,+16 or 47,XY,+16)
Trisomy 17 (47,XX,+17 or 47,XY,+17)	Trisomy 19 (47,XX,+19 or 47,XY,+19)	Trisomy 20 (47,XX,+20 or 47,XY,+20)
Trisomy 22 (47,XX,+22 or 47,XY,+22)	Triploidy (69,XXX, XXY or XYY)	Tetraploidy (92,XXXX or XXYY)

*Note*: These abnormalities can be detected by chromosome analysis.

**Figure 1:**
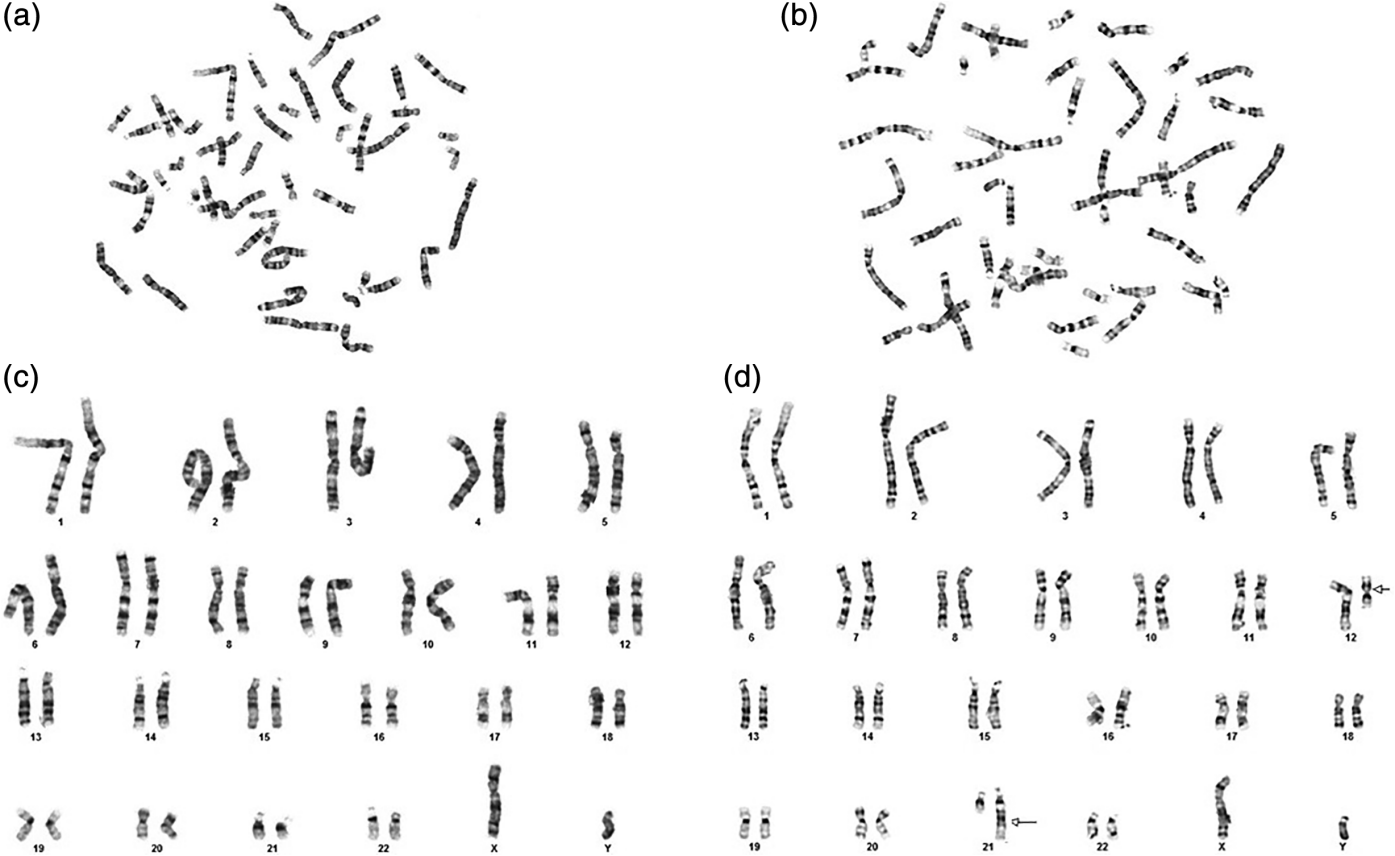
Chromosomes from a normal (a) and an abnormal metaphase (b) prepared by G‐banding technique. Karyotyping of the chromosomes form the normal cell (ISCN: 46,XY) is shown in (c), while karyotyping of chromosomes from the abnormal metaphase (ISCN: 46,XY,t(12;21)(q12;q21)) is shown in (d). ISCN stands for International System for Human Cytogenetic Nomenclature

Cytogenetics is a subspecialty of clinical genetics focused on detection of chromosomal abnormalities. Chromosomal abnormalities contribute to distinct and recognizable abnormal phenotypes. One of the most common tests to evaluate for chromosomal abnormalities ordered and performed in genetic laboratories is chromosome analysis, otherwise known as karyotyping. Chromosome analysis tests for numerical abnormalities (gain or loss of chromosomes), and structural abnormalities, such as partial deletions and/or duplications of DNA, balanced reciprocal translocations, Robertsonian translocations, para or pericentric inversions, balanced or unbalanced insertions, isochromosomes, marker, and ring chromosomes. The sample collected for this type of testing can vary from peripheral blood, skin fibroblasts, products of conception, amniotic fluid, chorionic villi, and so on. The samples are cultured under specific conditions and/or in the presence of different reagents to stimulate cell division and arrest of as many cells as possible in mitotic metaphase (one of the stages of the cell cycle). In metaphase, DNA is found in its most packed and condensed structure, forming visible and distinguishable units called chromosomes. Each cell, except for gametes, contains 46 chromosomes, 44 of which are pairs of autosomal chromosomes (22 pairs) and the sex chromosomes X and Y. There are different types of staining techniques to make chromosomes visible under the microscope for analysis, however the most frequently used technique is called G‐banding, which stands for Giemsa stained trypsin treated chromosomes. In G‐banded chromosomes dark bands represent gene‐poor regions and pale or lightly stained bands represent gene‐rich regions (Figure 1). The band pattern for each individual chromosome is well established and serves as a “measuring tool” to compare and distinguish extra or missing pieces at a resolution of approximately 5–10 megabase pairs in size.

Typically chromosome analysis is performed by analyzing 20 metaphase cells. In rare cases of mosaicism, two or more different cell lines are generated from abnormal cell division, one with the normal number of chromosomes and one or more with extra or missing chromosomes. When mosaicism is suspected such as in Turner syndrome, 30–50 cells are analyzed. This is also known as mosaicism study.

#### 2.1.1 Limitations

Genomic imbalances of approximately 5–10 Mb can be detected by chromosome analysis when the chromosome resolution of 550 bands is achieved. It is estimated that on average the sensitivity of detecting 5 Mb deletion or duplication is approximately 70%. Genomic imbalances of greater than 5 Mb in size could be missed, especially if there is no change in banding pattern. Fresh tissue is needed for chromosome analysis and typically cell culture is required, which in practical terms means that it can take up to 2 weeks (3–6 weeks for culturing fibroblasts from skin biopsy) to have the living and dividing cells needed to perform the analysis. In very rare cases, very few or no metaphase cells are available for analysis, which results in suboptimal studies. Furthermore, submicroscopic deletions or duplications, cryptic rearrangements, chromosomal mosaicism either of a whole chromosome or of subtle structural chromosome abnormalities may be missed. Abnormalities caused by molecular genetic mutations or environmental factors will not be detected by this method. Despite these limitations, a major advantage of chromosome analysis is that it provides whole genome assessment of both numerical and structural abnormalities in one single test.

## 3 MOLECULAR CYTOGENETIC ANALYSIS METHODS

### 3.1 Fluorescence in situ hybridization

**Figure 2:**
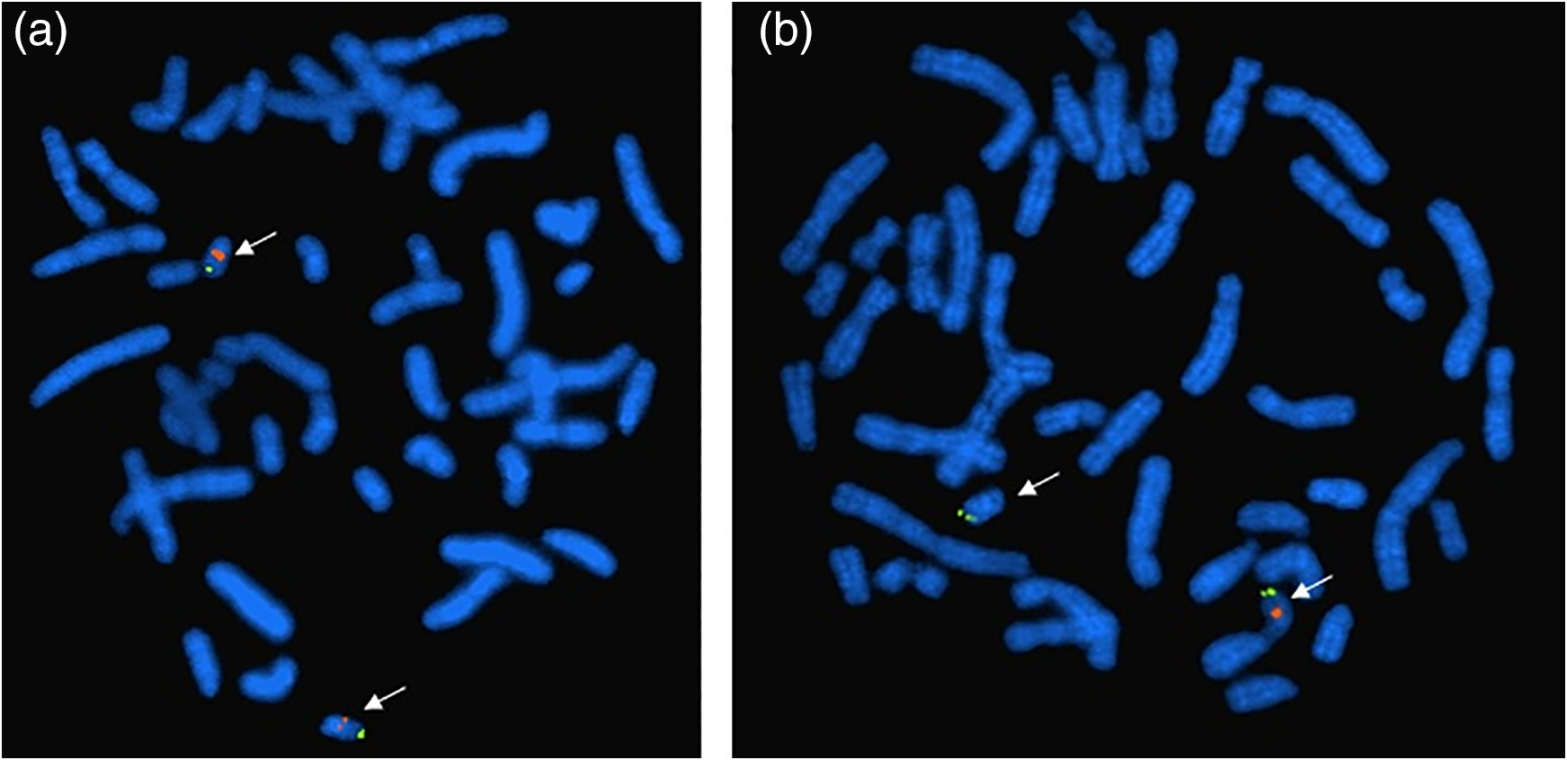
Centromere (green) and locus‐specific (orange) fluorescence in situ hybridization (FISH) probes are used to detect 22q11.2 deletion. (a) Normal; (b) a deletion is detected in cells from peripheral blood specimen by FISH with a probe specific for the 22q11.2 region. Probe is detected in only one of the two chromosomes 22, suggesting that a deletion is present. This finding is consistent with a diagnosis of DiGeorge syndrome or Velo‐Cardio‐Facial syndrome

**Figure 3:**
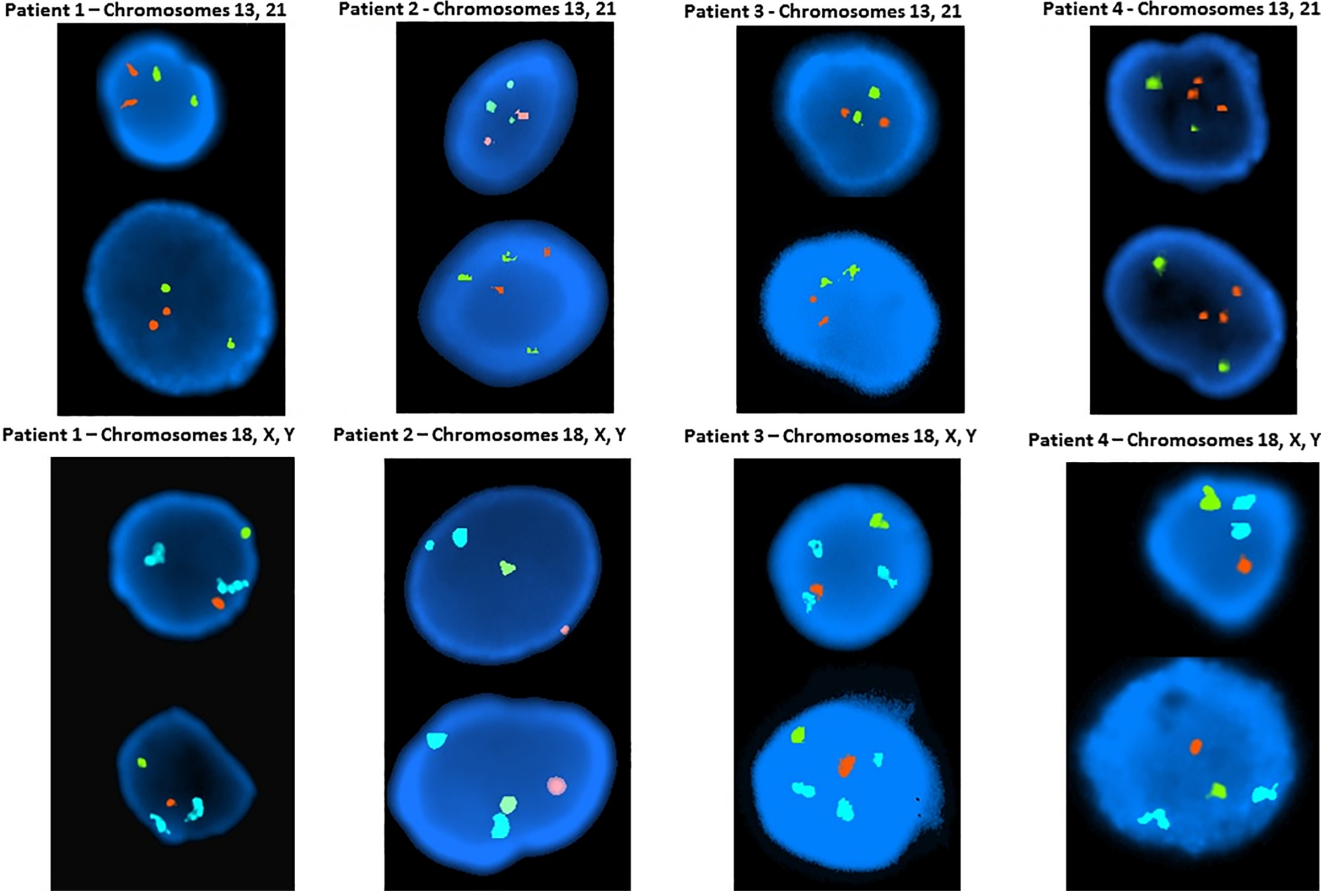
Fluorescence in situ hybridization (FISH) was used on uncultured interphase cells from amniotic fluid sample of four different male patients to detect the number of chromosomes 13 (green) and 21 (red) presented in the top panel. Additional interphase cells were examined to detect the number of chromosomes 18 (aqua), X chromosome (green), and Y chromosome (orange) present at the bottom panel. All FISH probes used in this assay are centromere specific probes. Patient 1 has the normal number of chromosomes tested, Patient 2 is positive for trisomy 13, Patient 3 is positive for trisomy 18, and Patient 4 positive for trisomy 21

Fluorescence in situ hybridization, commonly known as the FISH test, paved the way for the introduction of modern technology in the field of cytogenetics. FISH is a method in which fluorescently labeled probes bind or hybridize to specific complementary single stranded DNA or RNA sequences. After the probe hybridization, the cells or tissues undergo washing steps and the preparations are analyzed using a fluorescent microscope. Various probes can be labeled with specific fluorescent molecules with distinct excitation and emission characteristics. These applications allow simultaneous analysis of several probes including control probes. In the case of chromosome 22q11.2 deletion, associated with DiGeorge syndrome, two FISH probes are used simultaneously: the locus‐specific probe, which hybridizes to the commonly deleted region (emitting orange fluorescence) and the control probe that hybridizes to a specific region in the distal end of chromosome 22q arm (emitting green fluorescence; Figure 2). Normally, two orange and two green signals are observed. In the case of an abnormal test, one orange and two green signals are observed, indicating the absence or deletion of one copy of the 22q11.2 region associated with the syndrome. Currently, FISH probes and applications fall into two main categories, centromeric probes and locus‐specific probes. For example, the centromeric probes are used to determine the origin of ring or marker chromosomes and locus‐specific probes target genomic regions known to be associated with certain disease (Table 2). Additionally, FISH testing can be used for rapid detection of chromosome abnormalities in the context of prenatal diagnosis on interphase or nondividing cells (Figure 3).

**Table 2:** Known microdeletions and/or reciprocal microduplications, listed by chromosome number and location, from the beginning of the p arm (pter), descending to the end of the q arm (qter)

Microdeletion/microduplication syndromes			
Recurrent microdeletions		Recurrent microduplications	
Proximal 1q21.1	Thrombocytopenia‐absent radius (TAR) syndrome		
Distal 1q21.1 deletion	1q21.1 microdeletion (variable phenotype)	Distal 1q21.1 deletion	1q21.1 microduplication
3q29	3q29 microdeletion	3q29	3q29 microduplication (variable phenotype)
4p16.3	Wolf–Hirschhorn syndrome	4p16.3	4p16.3 microduplication syndrome
5p15.3	Cri‐du‐chat syndrome		
5q35	Sotos syndrome	5q35	5q35 microduplication, short stature/microcephaly
7q11.23	Williams syndrome	7q11.23	7q11.23 microduplication, autism
8p23.1	8p23.1 microdeletion syndrome	8p23.1	8p23.1 microduplication (variable phenotype)
15q11.2q13.1	Prader–Willi/Angelman	15q11.2q13.1	15q11.2q13.1 microduplication, susceptibility to autism
15q13.2q13.3	15q13.3 microdeletion syndrome	15q13.2q13.3	15q13.3 microduplication syndrome (variable phenotype)
16p13.11	16p13.11 microdeletion syndrome	16p13.11	16p13.11 microduplication (variable phenotype)
16p11.2	16p11.2 microdeletion, developmental delay, intellectual disability, and/or autism spectrum disorder	16p11.2	16p11.2 microduplication (variable phenotype)
17p13.3	Miller–Dieker syndrome	17p13.3	17p13.3 microduplication
17p12	Hereditary neuropathy with liability to pressure palsies (HNPP)	17p12	Charcot–Marie‐tooth type 1A
17p11.2	Smith–Magenis syndrome	17p11.2	Potocki–Lupski syndrome
17q11.2	Neurofibromatosis type 1 (NF1)	17q11.2	17q11.2 microduplication (variable phenotype)
17q12	17q12 microdeletion syndrome	17q12	17q12 microduplication
17q21.31	17q21.31 microdeletion/Koolen‐de Vries syndrome	17q21.31	17q21.31 microduplication
22q11.2	DiGeorge syndrome/Velocardiofacial syndrome	22q11.2	22q11.2 microduplication (variable phenotype)

Some of the major advantages of FISH studies compared to chromosome analysis is that they can be performed on nondividing (interphase) cells from fresh or aged samples, when the tissue is insufficient or unsatisfactory for chromosome analysis. In most cases, results are available within 24–48 hr from sample collection. FISH method provides a better resolution than chromosome analysis, but only for the regions targeted by FISH probes.

#### 3.1.1 Limitations of FISH

The information generated by FISH studies is very specific, limited to the genomic region(s) hybridizing with the probe(s) used. Single‐gene point mutations, small insertions/deletions, intragenic tandem duplications, and gains and losses of smaller than 30 kb in size will be missed, depending on the probe design.

### 3.2 Chromosomal microarray

The field of cytogenetics has been revitalized with the advent of newer molecular technologies like chromosomal microarray analysis (CMA) and single nucleotide polymorphism (SNP) array for whole genome analysis (Iafrate et al., 2004; Redon et al., 2006). These technologies have transformed the way geneticists practice laboratory medicine. Instead of viewing the entire world from a bird's eye view to identify different countries (the whole genome), with CMA we now have the technical capability to zoom into specific cities and, in some cases, even super‐zoom to street level.

Cytogenetic imbalances are a frequent cause of birth defects, developmental delay/intellectual disability, autism spectrum disorders, multiple congenital anomalies, heart defects, and other disorders that cannot be explained by aneuploidy alone (Girirajan, Campbell, & Eichler, 2011; Miller et al., 2010). Once it was recognized (by telomere analysis) that a lot of chromosome aberrations occur at the end of chromosomes, it was a matter of time to understand that structural aberrations were major contributors and accounted for a significant proportion of chromosomal related anomalies. This understanding and wealth of knowledge came from application of cytogenomic array methodologies, which are superior to the alternative methods in detecting copy number variants (CNVs) known as microdeletions or microduplications. Whole genome microarray analysis has rapidly been adopted by clinicians attempting to obtain a diagnosis for pediatric patients affected with idiopathic developmental delay and/or multiple congenital abnormalities (Kaminsky et al., 2011). CMA analysis has improved the diagnostic yield from 3–5% to 15–20%, providing answers to many genetic disorders previously described as “cause unknown” thereby reducing extensive work‐up of patients. To date, at least 100 copy number changes resulting in distinctive phenotypes have been reported in the literature, compared to a handful known in the past.

A significant change from the predecessor methods is the use of DNA for CMA, “bypassing” the need to culture live cells. Consequently, it improved the ability to analyze DNA from nearly any tissue, including samples that could not be cultured. Broadly, microarray assays can be designed as targeted or whole genome array. Microarray assays use multiple probes varying in number from thousands to more than two million distributed across the entire genome, thus facilitating the analysis of the entire genome at a high resolution. Signals from multiple consecutive probes are used to determine CNVs. On average CNVs of 500 Kb in size are detectable with high confidence and in probe‐dense regions, the assay can detect microdeletions as small as 25 Kb in size and microduplications of approximately 50 Kb in size. Nowadays, the majority of laboratories are performing oligonucleotide or SNP based arrays. Oligonucleotide‐based arrays are mainly designed to detect copy number variations in the genome by taking advantage of oligonucleotide probes of approximately 60 base pairs in length. The oligonucleotide‐based arrays allow flexibility in probe selection, which translates into a higher degree of freedom for the laboratories in customizing the array content and probe density. For example, a laboratory can design a targeted array with probes hybridizing to regions in the genome associated with recurrent microdeletions and microduplications (Miller et al., 2010; Weise et al., 2012) of well‐established disorders (Table 2). In whole genome oligonucleotide arrays, the probes are distributed throughout the genome. Some regions, such as the ones harboring haploinsufficient or triplosensitive genes, recurrent microdeletion/microduplication syndromes, subtelomeric and other regions of clinical relevance, are designed to have a high‐density probe coverage.

The SNP‐based platforms use a combination of shorter oligonucleotide probes (approximately 20–50 bp) and SNP‐detecting probes that are designed to hybridize to highly polymorphic SNPs across the entire genome. SNP‐detecting probes can also play a role in determining gains or losses of genomic material. In addition, these probes play a critical role in detecting allelic imbalances, segments of homozygosity that could indicate possible consanguinity or represent uniparental disomy (UPD), especially isodisomy if only the proband is tested. Heterodisomy detection requires a trio study that includes the proband and both parents (Tucker, Schlade‐Bartusiak, Eydoux, Nelson, & Brown, 2012). UPD occurs when both copies of a specific chromosome come from the same parent, instead of one copy coming from each parent. Serious clinical conditions affecting growth and development can result from this abnormal situation, particularly when chromosomes 6, 7, 11, 14, and 15 are involved (Shaffer et al., 2001).

Microarray analysis will detect benign CNVs that are commonly found in the population and reported in databases of normal variation (estimated to represent approximately 5–10% of CNVs; Girirajan et al., 2011). The higher the array resolution, the more of these variants will be detected and filtered out by the laboratories. The de novo CNVs or clinically relevant CNVs undergo an in‐depth analysis and can be reported out as benign, likely benign, variant of uncertain clinical significance, likely pathogenic or pathogenic. Laboratories are guided by standard guidelines in interpreting and reporting out CNVs, which are described in detail (Kearney, Thorland, Brown, Quintero‐Rivera, & South, 2011; South et al., 2013). Currently, the American College of Medical Genetics and Genomics (ACMG) and other professional medical organizations recommend chromosomal microarrays as the first test to investigate clinically relevant gains or losses of chromosomal material for individuals with multiple anomalies that are not specific to well‐delineated genetic syndromes, individuals with nonsyndromic developmental delay or intellectual disability, and individuals with autism spectrum disorders. Furthermore, the American College of Obstetricians and Gynecologists (ACOG) recommends chromosomal microarray as a first‐tier diagnostic test for pregnant mothers who undergo invasive testing in cases of suspected congenital anomalies (in place of conventional karyotype analysis) (Dugoff, Norton, & Kuller, 2016; Levy & Wapner, 2018; Phadke, 2014; Wapner et al., 2012). In general, and especially in the setting of prenatal care, it is highly important and recommended that families should be very carefully counseled by trained geneticists or genetic counselors regarding the positive results, more specifically regarding the positive results of recurring microdeletions/microduplications with very low penetrance due to the difficulty in predicting the clinical phenotypic outcome(Richardson & Ormond, 2018).

#### 3.2.1 Limitations

Genomic microarrays can only detect chromosomal imbalances. Balanced rearrangements, including paracentric or pericentric inversions, balanced insertions, balanced translocations, and Robertsonian translocations, cannot be detected by this method. In addition, low‐level mosaicism for unbalanced rearrangements or aneuploidies (less than 10–20% in the cell population), single‐gene point mutations that are relatively infrequent causes of abnormal phenotypes in the population (less than 1%), epigenetic modifications, and the contribution of environmental factors to the clinical phenotype are not detectable by microarray. Therefore, the assay does not exclude the diagnosis of a disorder solely on the basis of not identifying a copy number change in an individual's genome. The assay is relatively expensive compared to the above described methods; however, the benefits outweigh the costs, as the results are provided in a timely efficient manner and the overall diagnostic yield is estimated to be 10–20%, compared to 3–5% by karyotype analysis or FISH.

## 4 MOLECULAR GENETIC ANALYSIS METHODS

The human genome is slowly but constantly changing and these changes are important for the evolution of species. Variations found in the human genome can be predominantly categorized into two major classes: CNVs and SNPs. Our human genome consists of approximately 3,000,000 base pairs; and recent projects, such as The 1000 Genomes Project Consortium reported that they discovered a wide spectrum of genetic variation after performing whole genome and exome sequencing studies in 2,504 individuals from 26 different populations. In total over 88 million variants, 84.7 million SNPs, 3.6 million short insertions/deletions (indels), and 60,000 structural variants, were found (Auton et al., 2015). Yet, if we compare two different people's genome at the DNA base‐pair level, the genome is 99.9% the same and in the remaining 0.1% are the variants that make each one of us unique (Auton et al., 2015; Craig Venter et al., 2001). An even smaller number of these variants constitute the genetic changes that play a crucial role in human genetic diseases. The focus of molecular genetic diagnostic testing is to detect small variants, such as: single base‐pair changes, small deletions, duplications, or insertions, large intragenic rearrangements, intronic changes, or changes in copy number of triplet repeats. Expansion of triplet repeats is the main biological mechanism associated with trinucleotide repeat disorders. When the variants are associated with a disease, they are referred to as pathogenic variants or disease‐causing mutations. Molecular genetic laboratories use different techniques and protocols to examine heritable and de novo changes in the human genome to identify pathogenic variants. Herein, we will describe briefly the principles applied, possible outcomes, applications and limitations of the most commonly used methods in variant analysis: DNA fragment size analysis, restriction enzyme fragment length analysis, multiplex ligation‐dependent probe amplification, methylation polymerase chain reaction (PCR), Sanger sequencing, gene panel sequencing, and whole exome and whole genome sequencing. Another method commonly used by academic or commercial molecular genetics laboratories is targeted microarray analysis for exon level deletion/duplication, which is described in the chromosomal microarray section.

### 4.1 PCR (DNA) fragment size analysis

Polymerase chain reaction amplification is the first and basic step for many molecular analyses, from deletion studies to gene(s) sequencing. The main purpose of PCR amplification is to make multiple copies of a specified segment in human DNA, for further analysis. Designing a highly specific and unique pair of short, complementary sequences (approximately 19–27 bp in length) called primers (forward and reverse) flanking the region of interest is how the laboratories ascertain the amplification of the region of interest. PCR reactions include primers, DNA template, DNA nucleotides (the building blocks), buffers, and the DNA polymerase enzyme. PCR fragments can be visualized by mixing with specific gel dyes and performing gel electrophoresis, which separates DNA fragments according to their size. A DNA ladder run side by side with the samples will provide a very close estimate of the fragment sizes. The simplest application of what we just described in a clinical lab is Y chromosome deletion studies, which aim to detect deletions on the long arm of chromosome Y known to be associated with male infertility. Several bands are amplified by different primer pairs and their presence or absence is visualized by running the PCR amplified products alongside appropriate controls in an agarose gel.

**Figure 4:**
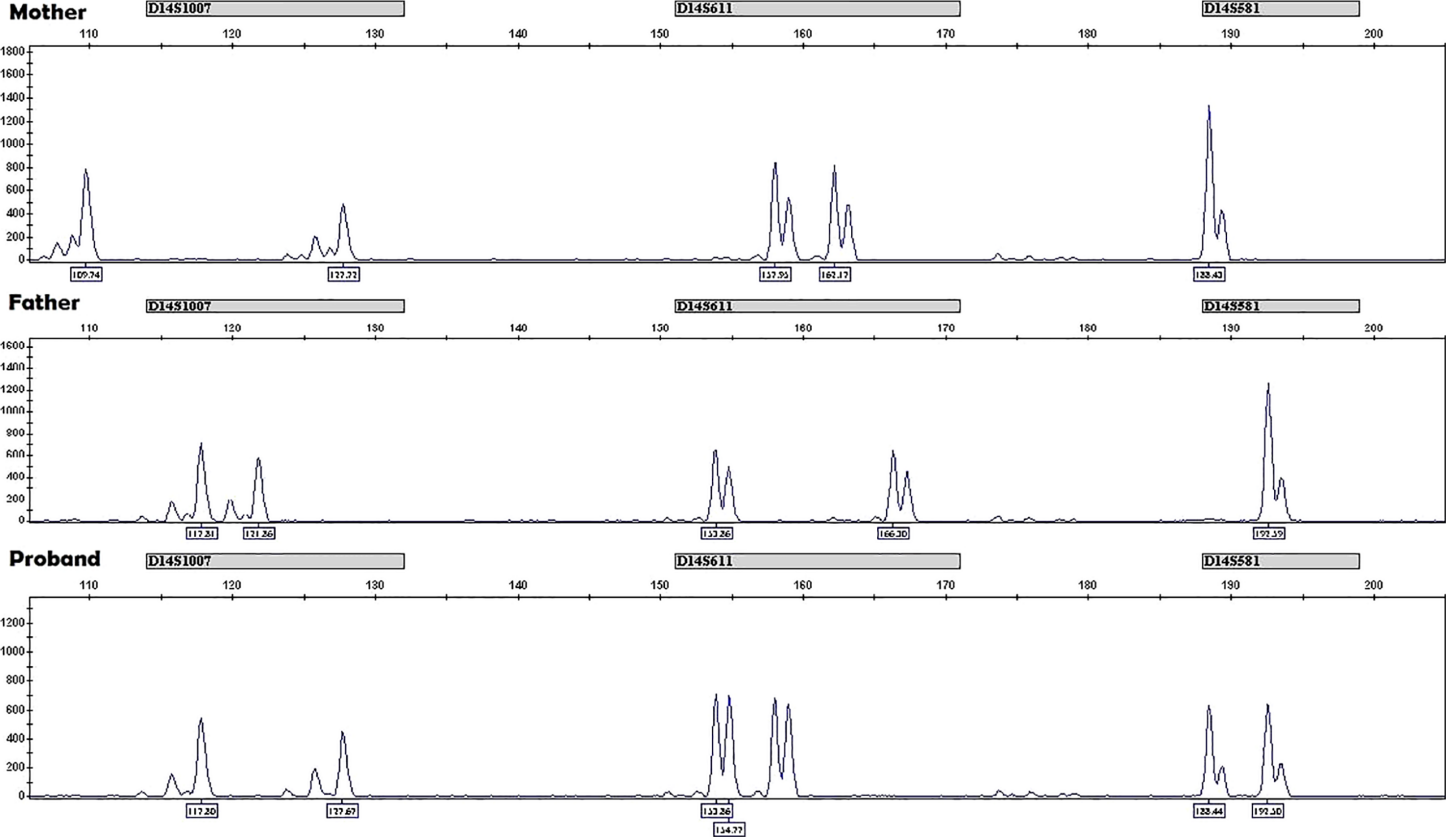
Multiplex quantitative fluorescence‐polymerase chain reaction (QF‐PCR) for short tandem repeat (STR) markers in a case of suspected UPD14. The image represents results for three out of nine polymorphic markers from chromosome 14 tested in DNA samples from proband and its parents (D14S1007, D14S611, and D14S581). The *x*‐axis shows length of PCR products in base pairs as determined by use of internal lane standard and the *y*‐axis shows the fluorescence intensity in arbitrary units. Biparental origin of the chromosome 14 in the proband can be inferred by analyzing inherited alleles and chromosome location of STR markers. For all three markers presented the proband has inherited one copy from the mother and one from the father

When UPD (Shaffer et al., 2001) is suspected or zygosity studies are needed to be performed for various reasons, the PCR fragment size analysis method uses the same concept, except for a few modifications that aim to improve the test's turnaround time, specificity and accuracy of results and interpretation. The forward primer in each primer pair is tagged by fluorophores (Figure 4). Gel electrophoresis is replaced by capillary electrophoresis. For example, in the case of suspected UPD of chromosome 14 associated with either maternal UPD of chromosome 14 (Temple syndrome) or the paternal UPD (Kagami–Ogata syndrome), the question the genetic test will attempt to answer is whether the two copies of chromosome 14 are coming from the same parent (Figure 4). Sequences of short tandem repeat markers (e.g., CA repeats) are amplified by PCR in the presence of fluorophore‐tagged forward primer (different fluorophores can be used to allow for more than two to four loci to be tested simultaneously). The target sequences amplified by PCR are distributed throughout chromosome 14. Capillary electrophoresis is run and an internal ladder control is used to analyze the signals/calls and determine the size of each individual fragment. Proband results are compared to each parent's results to determine if they are identical to either parent. Therefore, testing parental blood samples alongside child's or prenatal sample's DNA is highly important. Furthermore, it is strongly recommended that, at minimum, two fully informative loci should be obtained for diagnostic results. As any other method, this one comes with its own limitations, as negative results cannot rule out the presence of other genomic disease‐causing variants undetectable by this method.

### 4.2 Restriction enzyme fragment length analysis

These types of assays rely on using restriction enzymes with the ability to cut DNA at short, specific sequences called restriction sites. It can be used with the entire genomic DNA or a specific segment of DNA. Clinical laboratories can take advantage of the restriction enzymes' qualities to design cost and time efficient assays and observe whether an individual carries a variant in a gene for a disease that runs in his or her family. RFLP assay is performed to analyze human homeostatic iron regulator gene, also known as the *HFE* gene, for the two single base‐pair changes that lead to the disease‐causing amino acid changes (p.C282Y, p.H63D) known to be associated with hereditary hemochromatosis. This is a two‐step assay that utilizes a multiplex PCR amplification (Step 1) to simultaneously and with high specificity amplify the DNA segments of *HFE* gene followed by a restriction endonuclease digestion (Step 2). After the PCR products have undergone restriction endonuclease digestion, the fragments are examined by gel electrophoresis. Undigested fragments associated with the disease are bigger in size and migrate slower in the gel compared to digested products. One of the major disadvantages of this method is that it is not very robust and many samples cannot be analyzed in a short time.

### 4.3 Multiplex ligation‐dependent probe amplification

Multiplex ligation‐dependent probe amplification is a multiplex‐PCR based method that can detect abnormal copy numbers of genomic DNA or RNA, significantly improving detection rate in the laboratories for genetic diseases caused by (partial) intragenic gene deletions or duplications (Stuppia, Antonucci, Palka, & Gatta, 2012). It has several advantages compared to, and in some scenarios complements, other methods mentioned earlier in this article. Over 300 probe sets are commercially available, targeting different genetic conditions, including spinal muscular atrophy (SMA), DiGeorge syndrome, Duchenne muscular dystrophy, and so on. For the purpose of this article, we will discuss the principles and working steps for this method in the setting of testing for deletion and CNVs in the *SMN1* and *SMN2* genes associated with SMA. SMA is an autosomal recessive, neurodegenerative disorder resulting in progressive skeletal muscle weakness, generalized weakness, atrophy, and paralysis. SMA cases may be divided into three clinical categories based on the age of onset and the clinical course of the disease: Type I (severe), Type II (intermediate), and Type III (the milder form). Approximately 95% of the affected patients have homozygous deletion of exon 7 of the *SMN1* gene. The adjacent *SMN2* gene shares very high sequence homology to the *SMN1* gene. It has been shown that the number of *SMN2* copies modulates the clinical phenotype of SMA (Alías et al., 2018; Dubsky De Wittenau et al., 2012; Mailman, Heinz, Papp, et al., 2002).

The MLPA assay consists of several steps. The first one is the hybridization of specific probes to the genomic DNA. The SMA MLPA kit contains an *SMN1*‐specific exon 7 probe (274 nt) and the *SMN2*‐specific exon 7 probe (281 nt), which distinguish *SMN1* from *SMN2* by having their ligation site at the critical single nucleotide difference between these genes in exon 7. The second step is ligation of adjacent probes, followed by PCR amplification (Step 3). The unique characteristic of this assay is that the ligated probes will be the template for PCR reaction and amplify exponentially. This means that the PCR products are a direct measure of the number of the target sequences, in this assay of the copy numbers of exon 7 for both genes.

The PCR products are separated by capillary electrophoresis and the data analyzed by comparing the peak pattern obtained to that of the reference samples.

#### 4.3.1 Limitations

Although, the assay will accurately detect complete absence of at least exon 7 of the SMN1 gene in 95% of SMA patients, the remaining patients might have a single copy of the *SMN1* gene inactive due to a point mutation. MLPA assay cannot detect these cases. Also, normal polymorphic variations can affect probe binding and result in false positives.

### 4.4 Methylation PCR

**Figure 5:**
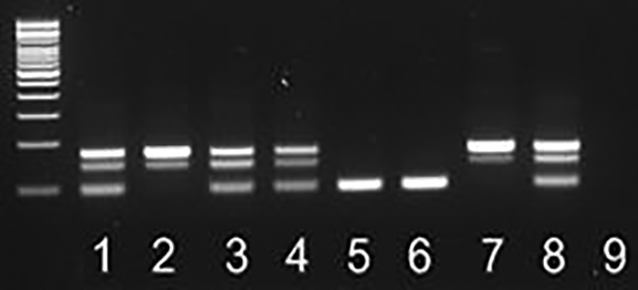
Agarose gel electrophoresis of methylation‐specific PCR (MS‐PCR) analysis of SNRPN locus. From left to right: DNA ladder with 500‐bp marker, the smallest band size is equal to 100 bp; 1, 3, and 4: normal patient samples; 2: patient DNA positive for PWS; 5: patient DNA positive for AS; 6: positive control for AS; 7: positive control for PWS; 8: controls for normal sample; 9: negative PCR control. AS, Angelman syndrome; PWS, Prader–Willi syndrome

DNA methylation pattern in CpG islands is an essential mechanism by which the cell regulates gene expression of imprinted genes. CpG islands represent long stretches of DNA within regulatory regions with a high G + C content and a high frequency of CpG dinucleotides compared to the whole genome. Imprinted genes are the ones whose expression is determined by the parent of origin (Kotzot, 2008). Prader–Willi syndrome (PWS) and Angelman syndrome (AS) are two clinically distinct neurodevelopmental genetic disorders that develop as a consequence of loss of expression of imprinted genes within this region (Cassidy, 1996). In patients with Prader–Willi syndrome, there is a loss of function of the paternal allele, while in patients with Angelman syndrome there is a loss of function of the maternal allele for several genes located on the proximal long arm of human chromosome 15 (15q11‐q13). Genetic testing is highly recommended to confirm the clinical diagnosis (Cassidy, 1996; Kotzot, 2008). Due to different mechanisms involved in causing Prader–Willi/Angelman, a number of cytogenetic and molecular tests can be ordered for the confirmation of these two disorders. Methylation‐specific PCR at the SNRPN locus is among the first diagnostic genetic tests performed and it will detect more than 99% of individuals with PWS and about 80% of individuals with AS. Methylation‐specific PCR relies on the fact that greater than 96% of the cytosine residues in the SNRPN locus are methylated on the maternal allele and none of them is methylated on the paternal allele. The initial step is a sodium bisulfite treatment of the proband's DNA, which converts unmethylated cytosine residues to uracil. Post‐sodium bisulfite treatment paternal and maternal copies of this region can be differentially amplified by PCR and yield PCR products of different sizes, 174 bp in size for the maternal allele and 100 bp for the paternal allele (Figure 5). Normal controls will have both bands amplified, PWS‐affected patients will have the maternal band, whereas AS‐affected patients will have the paternal band only.

#### 4.4.1 Limitations

methylation PCR will not detect AS cases caused by mutations, or in rare cases intragenic exonic deletions, in the *UBE3A* gene that account for about 10% of individuals with AS. Furthermore, this approach will confirm a diagnosis, but cannot provide further insight into the disease mechanism. Follow up studies as presented in detail in practice guidelines for the molecular analysis of PWS and AS by Buitin (2010) might be needed.

### 4.5 Single‐gene analysis by Sanger sequencing

**Figure 6:**
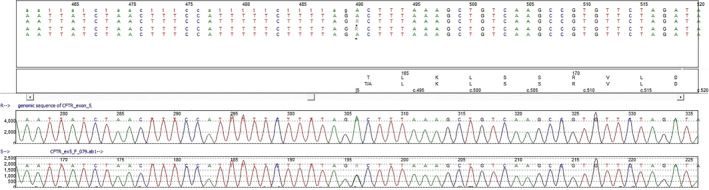
The electropherogram from Sanger sequencing analysis of a patient's DNA shows a nucleotide change from A to G (variant noted with an R), compared to the human reference genomic sequence. A heterozygous variant is detected, as each allele harbors a different nucleotide

Sanger dideoxy terminator DNA sequencing, broadly known as Sanger sequencing, is a laboratory technique used to interrogate genes' entire coding sequence for small disease‐causing variants, including single base changes, a few base‐pair deletions/duplications, and so on. It is considered the gold standard for detecting these small sequence changes and has been extensively previously described. As an example, this method can be applied to detect variants in all of the 27 coding exons of the *CFTR* gene associated with cystic fibrosis. Exons are the gene sequences that will end up being translated into protein by the translation protein complexes located in the cytoplasm. Pathogenic variants might affect the protein function, expression level (dosage), protein transportation to its final destination in the cell (example: membrane proteins), and so on. The first step in analyzing each exon's DNA sequence is PCR amplification, which is then followed by bidirectional sequence analysis. During this second step, PCR products are processed in a sequence analyzer through the process of capillary electrophoresis. Fluorescent DNA sequencing is performed by using fluorescently labeled ddNTPs. The sequencing reaction includes DNA template (PCR amplified DNA), unlabeled forward or reverse primer, buffer, the four dNTPs, the four fluorescently labeled ddNTPs, and DNA. Polymerase Fluorescent fragments are generated by incorporation of dye‐labeled ddNTPs and each different ddNTP (ddATP, ddCTP, ddGTP, or ddTTP) will carry a different color of dye. All terminated fragments (those ending with a ddNTP) will contain a dye at their 3′ end. Sequencing results are analyzed using software that permits direct visualization and will “spell” the DNA base pair and its position in the sequence (Figure 6). The end result is a sequence of DNA nucleotides that can be compared to the reference human genome to determine if any base‐pair changes are present. The genetics community is building comprehensive databases of variants identified in several disease‐causing genes, especially the ones that are associated with severe diseases manifesting in infancy or early childhood (Bean & Hegde, 2016). More than 1,700 variants of the CFTR gene have been identified to date; some are common, and others are rare variants and found in only a few people. Sanger sequencing is the “gold standard” confirmatory test for many variants discovered by next‐generation sequencing (NGS) assays (see next section).

#### 4.5.1 Limitations

Sanger sequencing is particularly useful for small types of mutations. However, other technologies are required for detection of large rearrangements or copy number variations, such as large deletions or duplications. Other limitations include low throughput, high cost, and very laborious and expensive when more than one gene needs to be tested. Generally, negative test results from gene sequencing do not rule out tissue specific or somatic mosaicism. More specifically, Sanger sequencing method is characterized by low sensitivity, with a limit of detection of approximately 10–15%, meaning that a mutation will not be detected if it is present in less than 10–15% of DNA molecules (Chin, da Silva, & Hegde, 2013; Davidson et al., 2012).

### 4.6 Next‐generation sequencing

Sequencing technologies have experienced rapid expansion and progression in the past few years, driven by the need for faster and more inclusive genetic testing (analyzing more than one gene at a time). Nowadays the sequencing platforms are capable of sequencing the whole human genome of a single cell through massive parallel sequencing or NGS (Yohe & Thyagarajan, 2017). Several applications of NGS technologies are moving into the clinical laboratories, with many more in the validation phase. Currently, three main levels of analysis can be performed by NGS: disease‐targeted gene panels, whole exome sequencing, and whole genome sequencing. The main steps of an NGS assay include DNA extraction, library preparation, target enrichment, sequencing, and data analysis. DNA can be extracted from any type of sample or tissue, including formalin‐fixed paraffin‐embedded tissue. The library preparation step will take the genomic DNA and break it into fragments, and then will add adapters to the fragments' ends in preparation for the sequencing step. Target enrichment is only performed for targeted exome sequencing and targeted gene panels and can be achieved by either hybridization of fragments of interest to complementary sequences or by PCR amplification. During sequencing, DNA fragments are first immobilized and then clonally amplified to generate a strong enough signal for detection. The sequencing step consists of sequencing by synthesis (similar to the principle applied to Sanger sequencing). When the sequencing process is repeated on both ends of the DNA fragment, it is referred to as “paired end reads”. Current sequencing platforms are reviewed in detail elsewhere (Yohe & Thyagarajan, 2017). The raw data undergo a very complex series of bioinformatic processes collectively called “pipeline” and variant interpretation. The variant interpretation step is simpler with targeted gene panels and it becomes more complicated as the portion of the genome being sequenced expands, mainly due to a higher probability of finding rare or novel variants. There are several guidelines published to facilitate and standardize variant calling and interpretation by different laboratories (Rehm et al., 2013; Richards et al., 2015).

*Disease‐targeted gene panels* investigate known disease‐associated genes. These panels have several advantages, including a greater sequencing depth (>500×), can be customized, identify SNPs, and insertions/deletions in genes of interest, are the most cost effective, and have better analytical sensitivity and specificity, compared to the other two. Increased depth coverage translates into higher confidence in making heterozygous calls and/or detecting mosaicism. Data storage is more manageable, which is a real challenge for laboratories, especially when considering whole genome sequencing.

*Whole exome sequencing* (*WES*) includes all protein coding regions of the genome. Although the exome represents only 1–2% of the whole genome, this portion of the DNA harbors approximately 85% of disease‐causing mutations known to date. Recent studies have reported a yield of approximately 20–25% in diagnosing previously undiagnosed rare disorders via WES. Sequencing depth is greater than 50×–100× and it can detect SNPs, insertions/deletions, and structural variation (SV). The diagnostic yield of exome sequencing ranges from 20 to 30%, meaning that exome sequencing will identify the disease‐causing variant in approximately 25% of the previously undiagnosed cases (Gahl et al., 2012; Lazaridis et al., 2016).

*Whole genome sequencing* (*WGS*) attempts to cover both coding and noncoding regions of the genomic DNA, including both nuclear and mitochondrial DNA. Adding the noncoding regions of genomic DNA provides information on SNPs, insertions/deletions, and SV involving noncoding regulatory RNAs, regulatory regions of gene expression (promoters, enhancers, and silencers), deep intronic regions involved in splicing, and so on. Sequencing depth coverage for this assay is greater than 30×. It is the most expensive of the three options, due to the high cost of data analysis and is not always covered by the medical insurance companies. Determination of pathogenicity of the variants detected by this method can be very challenging.

#### 4.6.1 Limitations

Although NGS sequencing is the most advanced technology in the medical genetic laboratories, it still is not an all comprehensive approach and significant limitations exist. Several areas in the genome, such as long repetitive sequences, are very difficult to sequence or analyze. Other limitations include the difficulty to interpret novel or rare variants due to lack of knowledge or absence of relevant functional tests, resulting in reporting these as variants of uncertain clinical significance. Many of the detected variants, including structural gene and copy number variation, will need to be confirmed by additional testing, thus adding to the cost and consequently increased anxiety in patients and families. Integration of genomic information into the medical care of patients has been another setback. These and other limitations not mentioned here will need to be addressed before NGS assays become a single method of detecting all clinically relevant genetic variants in the future.

#### 4.6.2 Evolution of WGS

For many infants born with rare and puzzling conditions, rapid diagnosis is critical, not only for diagnostic purposes, but also because it enables the clinical personnel to apply life‐saving interventions as soon as possible after birth. Birth defects and major structural anomalies affect approximately 3% of all pregnancies. Recently, a research study developed a WGS platform that makes it possible to obtain whole genome test results in a median time of about 20 hr by using computer systems able to perform automated phenotyping and data interpretation, tasks that normally require human intelligence (Bell, 2018; Clark et al., 2019).

## CONFLICT OF INTEREST

The authors declare no conflicts of interest relevant to this work.

## Data Availability

Data sharing is not applicable to this article as no new data were created or analyzed in this study.
